# Drug-related Halitosis: A Systematic Review

**DOI:** 10.3290/j.ohpd.a44679

**Published:** 2020-07-04

**Authors:** Hamed Mortazavi, Behrad Rahbani Nobar, Shervin Shafiei

**Affiliations:** a Professor, Department of Oral Medicine, School of Dentistry, Shahid Beheshti University of Medical Sciences, Tehran, Iran. Idea, hypothesis, discussion, proofread the manuscript.; b Dental Student, School of Dentistry, Shahid Beheshti University of Medical Sciences, Tehran, Iran. Systematic search, screening, data extraction, quality assessment, wrote introduction.; c Resident, Department of Oral and Maxillofacial Surgery, School of Dentistry, Shahid Beheshti University of Medical Sciences, Tehran, Iran. Screening, data extraction, materials and methods, quality assessment, proofread the manuscript.

**Keywords:** adverse drug events, adverse drug reactions, drug side effects, extra-oral halitosis, halitosis

## Abstract

**Purpose:**

Halitosis is an unpleasant breath odor which can be bothersome to individuals. Extra-oral halitosis is a type of halitosis caused by systemic conditions, bloodborne diseases, or pharmaceutical therapy. It is not related to local factors in the oral cavity. This systematic review aimed to identify the medications that can cause extra-oral halitosis.

**Materials and Methods:**

This study was conducted in accordance with the preferred reporting items for systematic reviews and meta-analyses (PRISMA). We searched online databases and also included hand searching to find relevant articles. Two authors independently performed the screening, data extraction and quality assessment of the included articles using the Cochrane Collaboration assessment tool.

**Results:**

Thirty-four studies met the eligibility criteria. The medications which can cause extra-oral halitosis were categorised into 10 groups: acid reducers, aminothiols, anticholinergics, antidepressants, antifungals, antihistamines and steroids, antispasmodics, chemotherapeutic agents, dietary supplements, and organosulfur substances.

**Conclusion:**

Pharmaceutical therapy is a potential source of extra-oral halitosis. This finding can help clinicians detect the probable causes of halitosis. Further studies are needed to definitely determine the role of various medications in causing extra-oral halitosis.

Adverse drug events are unintended injuries resulting from medication intake.^5,27^ The inevitable side effects of medications are among adverse drug events inherent to their specific properties. Medications can cause side effects in different parts of the human body. One such part is the oral cavity, in which several well-documented side effects can occur, e.g. those related to mucosa, salivary glands, periodontium, jawbones, teeth, sensory function, and taste.^31^ Dry mouth has been reported as the most frequent adverse drug event in the oral cavity^55^ and can contribute to halitosis.

Genuine halitosis is defined as an unpleasant breath odor which can have adverse psychological effects and hinder social communication.^22,32^ It may also indicate the presence of an underlying systemic condition such as diabetes mellitus or hepatic failure.^19^ A study concluded that an estimated 31.8% of adolescents and adults may have halitosis.^50^ Such a high prevalence rate can partly be explained by the fact that halitosis has several intraoral and extra-oral causes. The extra-oral causes are related to systemic and bloodborne conditions not related to the oral cavity itself.^41^ Drug-related side effects are an example of such etiologies, and are a common cause of treatment discontinuation by patients.^30,34^ Therefore, knowledge about drug-related side effects, e.g. halitosis, can help practitioners to devise measures to prevent their occurrence. This may, in turn, improve patients’ cooperation and quality of life.

Various studies have addressed halitosis and its pathogenesis. However, a detailed description of drugs that can cause halitosis has yet to be offered. A recent literature review^52^ identified nine medications and compounds that can cause halitosis. Routine drug therapy has also been shown to induce halitosis.^36,37,56^ However, to the best of the authors’ knowledge, no systematic review has attempted to identify the medications causing extra-oral halitosis. Prior knowledge about these medications can help clinicians in their decision making when prescribing medication or help them detect the probable causes of halitosis.

This study sought to identify the medications that can cause halitosis through a systematic review of the literature. In this process, it aimed to answer the following questions:

1. Which drugs can cause or contribute to extra-oral halitosis?
2. What dosage of prescribed medications can cause halitosis?
3. What are the routes of administration of such medications?
4. How prevalent is extra-oral halitosis in patients taking such medications?

## Materials and Methods

The methodology of this study conformed to the preferred reporting items for systematic reviews and meta-analysis (PRISMA) guidelines.^33^ The study was registered in the PROSPERO database under the registration number CRD42019129337.

### Eligibility Criteria

The PICOS elements were: population – patients in need of medications or healthy individuals; intervention – medications, pharmaceutical agents or dietary supplements; comparison – not using medications, pharmaceutical agents or dietary supplements, or using medications with a different dosage or a placebo; outcome – halitosis (bad breath).

### Inclusion Criteria

1. Clinical trials or cohorts
2. Patients must not have had halitosis at the onset of study
3. Patients must have received at least one medication during the study
4. Halitosis must have been reported as an individual side effect in numbers or percentages
5. If pseudohalitosis or halitophobia were present, genuine halitosis records must have been segregated from these entities
6. Halitosis that had originated from the systemic effects of the medication
7. Articles in English
8. Human studies

### Exclusion Criteria

1. Studies with a cross-sectional design, review, or pooled safety results design
2. Studies which had interventions that did not include drug therapy
3. Studies which had reported halitosis obscurely or in combination with other adverse events
4. Studies that did not mention the drug dosage
5. Studies that had combined the records of genuine halitosis, pseudo-halitosis, and halitophobia
6. Halitosis that had not originated from the systemic effect of the medication
7. Halitosis which could be directly linked to the effect of medication on the oral environment
8. Articles not in English
9. Animal studies

### Search Strategy

Five online databases – including PubMed, Scopus, ScienceDirect, Ovid, and Cochrane library – were searched for relevant published articles up to the end of February 2019. The search queries for PubMed were as follows: (drug* or drug* toxicity or drug* toxicities or adverse event* or adverse effect* or adverse reaction* or side effect* or side reaction* or medication*) AND (halitosis or oral malodor or oral malodour or bad breath or foul breath or fetid breath or odor* or smell*) AND humans [MeSH]. The search queries were changed accordingly when exploring other databases. We also searched relevant articles suggested by the online databases and the reference lists of the selected or relevant articles for additional data.

### Study Records

After removing duplicates, two authors (BRN and SS) independently performed title and abstract screening. Full-text screening was reserved for articles which could not be screened promptly by their titles or abstracts. The same authors extracted the following data from the selected articles if present: author(s) name(s), year of publication, medication name, pharmaceutical group, subgroup, dosage, sample size, number and percentage of patients with halitosis, significance level/p-value, type of adverse effect assessment, and the present disease. Cohen’s kappa was used to determine the level of agreement between the two authors at this stage. The two authors discussed their disagreements concerning the study selection and data extraction with the third author (HM) and resolved them entirely.

### Quality Assessment

Two authors (BRN and SS) independently evaluated the risk of bias (RoB) of the selected studies individually using the Cochrane Collaboration assessment tool. The bias domains were evaluated and the overall RoB for each study was specified. The third author (HM) resolved the disagreements between the first and second authors regarding RoB.

## Results

**Fig 1:**
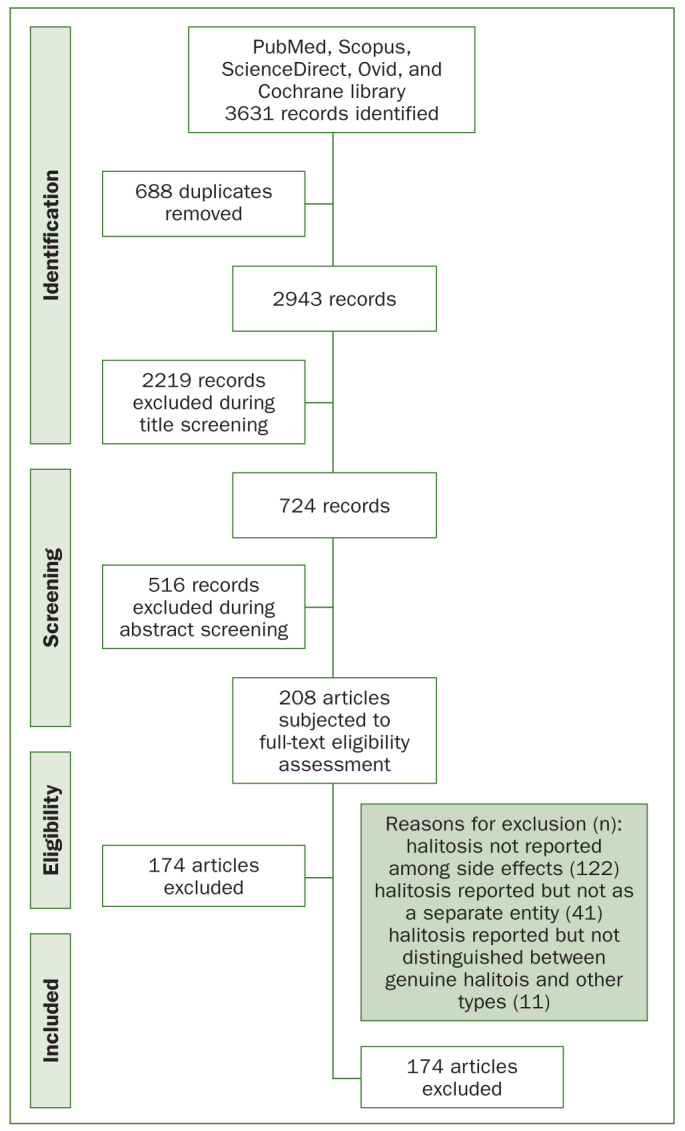
Study selection flowchart.

Figure 1 shows the flowchart of the search strategy and selection process of articles. After searching the databases and removing the duplicates, 2943 records were identified. After title and abstract screening, we assessed 208 articles by evaluating their full texts according to the eligibility criteria. Finally, 34 articles were included in this systematic review. The level of agreement between the two authors in the study selection and data extraction phase was 0.94.

Eighteen studies had low RoB, ten were unclear and six were found to have a high RoB. Table 1 shows the RoB of the included studies in terms of each bias domain. Studies included in this review evaluated a total of 30,736 patients, and a total number of 1932 (6.2%) cases of drug-related halitosis were reported in the trials. The administration routes of the medications widely varied and included oral intake, eye drops, topical application, subcutaneous injection, intradermal injection, intravenous injection, and intranasal administration. Some studies employed more than one route to administer the medication(s). Halitosis was assessed either by objective methods such as organoleptic testing and molecular evaluation of the exhaled air or subjectively according to patient complaints of bad breath. One study combined both methods,^49^ 11 studies used objective measures and 21 assessed halitosis subjectively. The rest of the studies did not explicitly state their assessment method. In Table 2, the studies are listed by alphabetical order of pharmaceutical group, presenting study summaries and overall RoB, as follows:

**Table 1:** Risk of bias of the included studies

Author and year	Selection bias	Performance bias	Detection bias	Attrition bias	Reporting bias	Other bias	Overall bias
Businger et al 1989	unclear	low	low	low	low	NA	unclear
Dubinsky, Gray 2006	unclear	unclear	unclear	low	low	NA	unclear
Besouw et al 2012	unclear	unclear	unclear	low	low	NA	unclear
Besouw et al 2007	low	low	low	low	low	NA	low
Ahlenstiel-Grunow et al 2016	low	low	low	low	low	NA	low
Corcos et al 2006	low	low	low	low	low	NA	low
Stern 1997	high	high	high	low	low	NA	high
Hurley et al 2006	unclear	unclear	high	low	low	NA	high
Volz et al 1997	unclear	low	low	unclear	low	NA	unclear
Purkins et al 2003	low	low	unclear	low	low	NA	unclear
Sibbald et al 1986	unclear	unclear	unclear	low	low	NA	unclear
Mosaffa-Jahromi et al 2016	low	low	low	low	low	NA	low
Baker et al 2014	low	low	low	low	low	NA	low
Ramanathan et al 2011	low	low	low	low	low	NA	low
Ramanathan et al 2010	low	low	low	low	low	NA	low
Ramanathan et al 2007	unclear	unclear	unclear	low	low	NA	unclear
Flaig et al 2006	low	low	low	low	low	NA	low
Christensen et al 2014	low	low	low	low	low	NA	low
Elwakeel and Hazaa.2015	low	low	low	low	low	NA	low
Cohen et al 2014	low	unclear	unclear	low	low	NA	unclear
Freeman and Sinha 2007	low	low	unclear	low	low	NA	unclear
Belluzi et al 1994	unclear	high	unclear	unclear	unclear	NA	high
Caniato et al 2006	unclear	high	unclear	low	low	NA	high
Dunn and Taylor 2011	low	low	low	low	low	NA	low
Asfour et al 2008	low	low	low	low	low	NA	low
Faikh et al 2006	low	low	low	low	low	NA	low
Ozkaya-Bayazit et al 1998	high	high	high	low	low	NA	high
Paul 1967	high	high	high	unclear	unclear	NA	high
Bookman et al 2004	low	low	low	low	low	NA	low
Shainhouse et al 2010	low	low	low	low	low	NA	low
Roth and Shainhouse 2004	low	low	low	low	low	NA	low
Tugwell et al 2004	low	low	low	low	low	NA	low
Baer et al 2005	low	low	low	low	low	NA	low
Barrou et al 2004	unclear	unclear	unclear	low	Low	NA	unclear

**Table 2:** Summaries of the included studies

Pharmaceutical group	Author(s)	Subgroup	Drug (N)	Dosage (N)	Administration	Sample size	Halitosis (%)	Significance/p-value	Type of adverse effect assessment	Disease	Risk of bias
Acid reducers	Businger et al 1989	H2 histamine receptor antagonist	Ranitidine	150 mg q.d	Oral	110	1 (0.9%)		Subjective (patient report)	Duodenal ulcer relapse prevention	Unclear
Aminothiols	Dubinsky, Gray 2006		Cysteamine (Cystagon)	10 mg/kg per day (increasing doses by 10 mg/kg per day weekly until 40 mg/kg per day)	Oral	8	8 (100%) (20 mg/kg per day or higher)		Objective (hydrogen sulfide oral odor)	Huntington’s disease	Unclear
	Besouw et al 2012		Enteric coated cysteamine bitartrate (RP103)	1.2-1.5 g/m^2^	Oral	4	4 (100%)		Objective (dimethyl sulfide and methanethiol breath measurements)	Cystinosis	Unclear
	Besouw et al 2007		Cysteamine	15 mg/kg once	Oral	8	8 (100%)		Objective (dimethyl sulfide and methanethiol breath measurements)	Cystinosis	Low
	Ahlenstiel-Grunow et al 2016		Immediate release cysteamine, extended release cysteamine	1.2 g/m^2^ per day, 0.9 g/m^2^ per day	Oral and eye drop	12	6 (50%) 2 (16.6%)		Subjective (self-breath assessment)	Cystinosis	Low
Anticholinergics	Corcos et al 2006	Muscarinic antagonists	Oxybutynin	5, 10, 15 mg/day	Oral	77-77-83 respectively (total=237)	6 (8%)-10 (13%)-8 (10%) respectively		Subjective (patient report)	Urge urinary incontinence (UUI)	Low
	Stern 1997		Glycopyrrolate	40-175 µg/day q.d	Oral	22	1 (4.5%)		Subjective (patient report )	Drooling	High
Antidepressants	Hurley et al 2006	SSRI	Duloxetine	20-80	Oral	958	5 (0.5%)	0.062	Not mentioned	Women with stress urinary incontinence	High
	Volz et al 1997	TCA	Imipramine	50-75 mg b.i.d (total dose of 100-150 mg/day)	Oral	96	1 (1%)		Subjective (patient report)	Major depression	Unclear
Antifungals	Purkins et al 2003	Triazole + protease inhibitor	Voriconazole + Indinavir	200 mg BID (17 days) -800 mg TID (last 10 days)	Oral	9	1 (11%) (subject dropped out at day 10)		Objective (examination)	Healthy subjects	Unclear
Antihistamines, steroids	Sibbald et al 1986		Beclomethasone diproprionate (BDP), or astemizole (AST)	2-4 puffs/nostril b.i.d, 10-30 mg daily	Intranasal, oral	50	9 (18%), 7 (14%)		Objective and subjective	Perennial rhinitis	Unclear
Anti-spasmodics	Mosaffa-Jahromi et al 2016		Colpermin (peppermint oil)	187 mg/capsule t.i.d	Oral	38	5 (13.1%)	0.03	Subjective (patient questionnaire)	Irritable bowel syndrome	Low
Chemotherapeutic agents	Baker et al 2014	Tumor growth inhibitor	PX-12	150-450 mg/m^2^ diluted with 5% dextrose solution	24-h infusion	18	1 (5.5%)		Objective (pungent breath odor detection)	Gastrointestinal malignancies	Low
	Ramanathan et al 2011		PX-12	300-400-500-600 mg/m^2^	72-h infusion	14	7 (50%)		Objective (garlic like breath odor detection)	Advanced or metastatic cancers, lymphoma, refractory tumors	Low
	Ramanathan et al 2010		PX-12	54,128 mg/m^2^	3-h infusion daily for 5 consecutive days	16	28%, 56%		Objective (expired air metabolite assessment)	Advanced pancreatic cancer	Low
	Ramanathan et al 2007		PX-12	9-300 mg/m^2^	1-3 hours of infusion daily for 5 consecutive days	38	100%		Objective (organoleptic and expired air metabolite assessment)	Advanced solid tumors	Unclear
	Flaig et al 2006		Silybin phytosome	2.5-20 g daily in three divided doses (increasing doses until maximum tolerable dose was found: 13 g/day	Oral	13	2 (15%)		Subjective (patient report)	Adenocarcinoma of prostate	Low
Dietary supplements	Christensen et al 2014		Rosehip powder + vitamin C	4500, 4500, 2250 mg/day in 6 capsules +80 mg one daily	Oral	150 in total 50-50-50	21 (14%) in total 3 (6%)-8 (16%)-10 (20%)		Subjective (symptoms)	Osteoarthritis of the knee	Low
	Elwakeel and Hazaa 2015	Fish oil	ω3 PUFA + Aspirin	1 g t.i.d + 75 mg q.d	Oral	20	13 (65%)		Subjective (patient report)	Chronic periodontitis and type II diabetes mellitus	Low
	Cohen et al 2014	Fish oil + vitamin	Omega-3 PUFA + vitamin E in one capsule placebo: olive oil + vitamin E	615 mg + 15 IU t.i.d 15 IU t.i.d	Oral	173 (162 according to the study), 160	5 (2.8%) (3.1% according to the study),4 (2.5%)		Subjective (patient report)	Perimenopausal and postmenopausal women	Unclear
	Freeman and Sinha.2007	Fish oil	Omega-3 PUFA placebo: corn oil + 1% fish oil	1.84 g/day	Oral 4 divided doses	23,36 A total of 59 patients	3 (13%), 2 (5.5%), 5 (8.4%) in total		Subjective (patient report)	Pregnant women, postpartum women	Unclear
	Belluzi et al 1994		Fish oil derivative purepa (40% EPA + 20% DHA), conventional fish oil	500 mg, 1 g capsules × 12 times daily (6 weeks)	Oral nine times daily, oral twelve times daily continued for 6 weeks	20 (10,10)	5 (50%), 6 (60%)		Not specified	Crohn’s disease	High
	Caniato et al 2006		Fish oil eicosopentaenoic acid + docosahexaenoic acid	3 g (1.8+1.2)	Oral divided by two daily doses in the form of 10 capsules daily	28	43%		Subjective	Schizophrenia or schizoaffective disorder	High
	Dunn and Taylor 2011	Selenium + vitamin	Vitamin E, l-Selenomethionine, vitamin E + l-Selenomethionine	Vitamin E: 400 mg/day, l-selenomethionine: 200 µg/day	Oral	8737-8752-8703	493 (5.6%) – 503 (5.7%) – 531 (6.1%)	NA-NA- p<0.05	Not specified	Healthy men	Low
	Asfour et al 2008	Selenium	Sodium selenite	0.2 mg/kg per day q.d	Oral	20	90%		Objective (garlic-like breath odor)	Patients with non-Hodgkin’s lymphoma (NHL) of intermediate and high grade who received concurrent chemotherapy	Low
	Faikh et al 2006		Selenomethionine amongst other anticancer and corticosteroid drugs which were not related to the outcome	2.2 mg/day	Oral	10	6 (60%)		Objective (garlic-like odor)	Metastatic or unresectable solid tumor	Low

- **Acid reducers:** These medications are used to treat gastric upset caused by excessive acid production. One study with unclear RoB showed that treatment with 150 mg/day ranitidine resulted in halitosis in one out of 110 patients.^12^
- **Aminothiols:** Cysteamine is used for the treatment of patients diagnosed with cystinosis or Huntington’s disease. In the surveyed studies, cysteamine doses of 15 mg/kg and higher^8,17^ or 0.9 g/m^2^ to 1.2 g/m^2^ caused halitosis in up to 100% of the patients.^1,9^ Two of the studies had low RoB,^1,8^ and two other studies had an unclear RoB.^9,17^
- **Anticholinergics:** Oxybutynin and glycopyrrolate are used to regulate or inhibit the parasympathetic system. Doses of 5 to 15 mg/day and 40 to 175 µg/day for the respective medications caused halitosis in 15.5% and 4.5% of the subjects, respectively.^16,51^ The RoB for these articles was unclear and high, respectively.
- **Antidepressants:** Imipramine, which belongs to the subclass of tricyclic antidepressants, caused halitosis in 1% of the subjects in a reviewed study. However, the RoB of this study was unclear.^54^
- **Antifungals:** The results of one study showed that antifungal treatment with voriconazole and indinavir resulted in halitosis in one out of nine patients. The RoB of this study was unclear.^42^
- **Antihistamines and steroids:** Antihistamines and steroids are well-known anti-inflammatory drugs. The results of an open crossover study utilising astemizole and beclomethasone dipropionate demonstrated that halitosis occurred in 14% and 18% of the group population, respectively. The RoB of this study was unclear.^49^
- **Antispasmodics:** Antispasmodics are used to relax muscles and inhibit muscle spasms. A variety of regions can be targeted with these medications in the human body, including the muscles of the gastrointestinal system. Peppermint oil (187 mg/capsule) administered three times daily caused halitosis in 13.1% of the patients diagnosed with irritable bowel syndrome.^35^ The RoB of this study was low.
- **Chemotherapeutic agents:** PX-12 is a tumour growth inhibitor used for the treatment of cancer patients mainly through infusion. Doses of 9 to 600 mg/m^2^were administered in the reviewed studies and halitosis was observed in 5.5% to 100% of patient populations.^4,43-45^ Three of these studies had a low RoB,^4,43,45^ while one had an unclear RoB.^44^
- **Silybin-phytosome:** Silibinin is another agent that inhibits tumour growth. Oral administration of one of its complexes, silybin-phytosome, at a dosage of 2 to 13 g daily resulted in halitosis in 15% of the trial’s population.^23^ The RoB of this study was low.

### Dietary Supplements

#### Fish oil

Fish oil contains omega-3 polyunsaturated fatty acids which can prevent cardiovascular diseases and other inflammatory conditions. Five reviewed studies administered doses of 1.84 to 12 g/day which resulted in halitosis in 3.1% to 60% of the study populations.^7,13,15,20,24^ The dosage of fish oil directly correlated with the incidence of halitosis. One of these studies had a low RoB,^20^ two had unclear RoBs,^15,24^ and two others had high RoBs.^7,13^

#### Rosehip powder

Rosehips have been traditionally used for their anti-inflammatory properties. A study conducted on 150 patients diagnosed with osteoarthritis of the knee reported that oral administration of 2250 to 4500 mg/day rosehip powder in combination with 80 mg/day vitamin C resulted in halitosis in 14% of the trial’s population.^14^ This study had a low risk of bias.

#### Selenium

Selenium’s anticancer properties are well documented in the literature. A study by Dunn and Taylor^18^ evaluated the cancer prevention effects of selenium (as l-selenomethionine) and vitamin E individually and in combination with each other. The authors concluded that while halitosis occurred in all the 3 groups, drug-induced halitosis in the selenium+vitamin E group was statistically significant (6.1% of the group). Other studies regarding selenium (as selenomethionine and sodium selenite) reported halitosis in 60% to 90% of patients, respectively.^2,21^ These three studies had low RoBs.

#### Organosulfur substances

Dimethyl sulfoxide (DMSO) is used for a variety of conditions such as amyloidosis and osteoarthritis. It is used either solely or as a carrier for various medications or materials such as diclofenac and cultured cells.^3,6,10,38,40,46,47,53^ Five of these studies had low RoBs,^3,10,46,47,53^ two had high RoBs,^38,40^ and one had an unclear RoB.^6^ The maximum and minimum rates of drug-induced halitosis in the studies were 100% and 0.6%, respectively. Interestingly, a study conducted by Tugwell et al^53^ found no significant difference in drug-induced halitosis between the two groups that applied different concentrations of DMSO to the skin.

It is worth mentioning that the use of substances such as garlic, onion, and alcohol can also result in extra-oral halitosis. However, because these substances were not used as a medication in the reviewed studies, we did not include them in our systematic review.

## Discussion

This systematic review sought to quantitatively assess the adverse effects of various medications on the breath by measuring the sample size and the incidence of halitosis in the treatment groups. Thirty-four articles were evaluated and various pharmaceutical groups and treatment modalities were identified.

Although we identified various pharmaceutical groups as potential sources of drug-related halitosis, the results of these studies must be interpreted with caution because their designs, RoB, and confounding factors affect halitosis assessment. One confounding factor is the oral cavity itself. A number of intraoral and extra-oral factors can cause halitosis.^41^ The studies included in this review were mainly medical studies intended to assess the efficacy of medications to improve the patients’ medical conditions, which were sometimes life-threatening. Thus, it can be assumed that oral hygiene measures might not have been emphasised to the extent they should have been, so that inadequate oral hygiene may also have contributed to halitosis in some cases. Furthermore, the possible intraoral effects of some of these drugs, such as ranitidine,^28^ oxybutynin,^26^ glycopyrrolate,^39^ duloxetine,^48^ imipramine,^25^ PX-1243, which can facilitate intraoral halitosis through dry mouth, must not be overlooked.

Although the studies reported halitosis as a potential side effect of the medication, most of their assessments were based on subjective measures (patient reports, questionnaire). Although subjective assessment of potential side effects is important in maintaining the patients’ quality of life, such evaluations should be complemented by a thorough objective assessment. However, only one study performed both a subjective and objective evaluation.

Systemic diseases can also cause halitosis through systemic pathways. One example is gastrointestinal diseases, which can result in events such as bloating, belching, or vomiting and therefore contribute to halitosis.^29^ Statistical methods used in these studies to analyse the adverse effects are another point of concern; most studies did not report the p-values for pairwise comparisons of halitosis. Thus, statistical significance could not be assessed and a meta-analysis was not feasible. See below for a more detailed description of the studies.

Two of the studies^16,51^ evaluated the effect of oxybutynin and glycopyrrolate anticholinergic medications. They reported halitosis as an adverse effect of these medications. Although a possible explanation for this was not provided, it can be assumed that the antisialogogic properties of these medications could have contributed to halitosis. Considering the RoB of these studies, their results must be interpreted with caution.

The results of some other studies were more reliable. Mosaffa-Jahromi et al^35^ found that the use of peppermint oil (Colpermin) in a group of patients with irritable bowel syndrome resulted in halitosis, with a statistically significant difference was found between those taking and not taking the medication. However, no explanation was presented for the potential cause of this side effect.

It is well documented that PX-12, a chemotherapeutic agent, can cause halitosis,^4,43-45^ so much so that PX-12 infusion can cause halitosis within minutes of infusion.^44^ Although the infusion took place in negative air-pressure rooms equipped with filters, a garlic-like odor was noted on patients’ breath.^45^ This odor was caused by exhalation of 2-butanethiol secondary to PX-12 metabolism.^45^

DMSO is an odorless compound used either solely or as a carrier for other medications such as diclofenac. A study by Ozakaya-Bayazit et al^38^ found that topical application of DMSO caused halitosis in all patients. This finding was attributed to the exhaled dimethyl sulfide, one of the metabolites of DMSO. Other studies found that the use of DMSO as a carrier for diclofenac can also cause halitosis, in both the treatment and the placebo carrier groups.^3,6,46,53^ However, it seems unlikely that diclofenac’s metabolism produces an odor similar to that of DMSO.

Fish-oil supplements are another possible source of halitosis. A study by Elwakeel et al^20^ found that oral administration of fish oil resulted in patients reporting fish-scented bad breath. However, this halitosis was mild and the treatment was continued in spite of it. Comparing the methodology and the results of the reviewed studies, it is hypothesised that the dosage of the administered fish oil can be correlated with the induced halitosis. Further studies are needed to clarify this matter.

Selenium is another potential source of halitosis. In the reviewed studies,^2,18,21^ selenium was used in the form of two complexes, i.e. selenomethionine and sodium selenite. A study conducted by Asfour et al^2^ showed that halitosis occurred in 90% of the trial’s patients. However, halitosis may have been enhanced by the fact that these patients were receiving concurrent chemotherapy. Another study by Faikh et al^21^ reported halitosis in 60% of cancer patients after administering selenomethionine. Interestingly, the study by Dunn and Taylor^18^ found that combined selenomethionine and vitamin E administration resulted in more halitosis cases compared with administering them singly. This finding was statistically significant and warrants further studies about the possible effects of vitamin E on halitosis.

The classification of medications which were included in this systematic review is intended to help clinicians detect halitosis and distinguishing between its types. Interestingly, when the metabolism of a medication alters the breath odor, it may also change the patient’s body odor.^10,11^ However, this phenomenon cannot be generalised to all medications, and several factors can hinder the clinician’s judgment including the effect of other medications on body odor. Thus, the reliability of this method needs to be investigated in future studies.

Clinicians should take note of the medications that can cause extra-oral halitosis and, if pragmatic, prescribe medications that do not cause halitosis or cause it to a lesser extent. This, in turn, can lead to improved quality of life outcomes. This knowledge can also help dentists and physicians determine the cause of newly emerged halitosis and save time and costs, leading to better patient cooperation.

Definite conclusions about the dosage or administration routes could not be drawn because of the diverse methods these studies used and a lack of studies comparing different doses and administration routes. The limitations of this systematic review were the lack of quantitative analysis and exclusion of studies which did not provide quantitative data regarding halitosis cases. This could have excluded some medications which can cause halitosis. Nevertheless, the authors of this review suggest that future trials utilise standardised protocols designed to evaluate adverse effects of medications on the oral cavity, including but not limited to halitosis. Further studies are needed to determine the extent of the capability of medications, systemic diseases, and other possible factors in inducing extra-oral halitosis and the specific pathways responsible for this adverse event.

## Conclusion

Clinicians should be aware that extra-oral halitosis may be caused by the use of medications such as cysteamine, ranitidine, oxybutynin, glycopyrrolate, imipramine, astemizole, beclomethasone diproprionate, Colpermin, PX-12, sylibin-phytosome, fish oil, selenium, vitamin E, DMSO, and diclofenac. However, due to confounding variables and high risks of bias across the studies, these findings must be viewed with caution.
